# SGLT1 and SGLT2 Modulation in Antidiabetic Therapy—Comparative Insights into Gliflozins and Natural Compounds Resveratrol and Viniferin

**DOI:** 10.3390/ph19030376

**Published:** 2026-02-27

**Authors:** Diana Petra Matei, Alina Dușanca Ghișe, Liliana Cărpinișan, Ioan Huțu, Adrian Stancu, Kalman Imre, Eugenia Dumitrescu

**Affiliations:** Faculty of Veterinary Medicine, University of Life Sciences “King Mihai I” from Timisoara, 300645 Timisoara, Romania; diana.matei@usvt.ro (D.P.M.); lilianacarpinisan@usvt.ro (L.C.); ioanhutu@usvt.ro (I.H.); adrianstancu@usvt.ro (A.S.); kalmanimre@usvt.ro (K.I.); eugeniadumitrescu@usvt.ro (E.D.)

**Keywords:** SGLT inhibition, antidiabetic therapy, resveratrol, viniferin, gliflozins

## Abstract

Glucose transport dysregulation plays a central role in pathophysiology of diabetes mellitus. Synthetic agents like sotagliflozin or canagliflozin have been discovered recently and are currently used as antidiabetic therapy, providing great efficacy in modulation of glucose transport. However, the search for additional compounds with antidiabetic potential continues, as researchers aim to identify new molecules that may complement or enhance existing therapies. Numerous plant-derived compounds are currently under investigation and have demonstrated promising effects, while others remain far less studied yet could hold meaningful potential. In this context, resveratrol and its oligomers, including ε-viniferin, have gained attention due to promising in vitro findings, particularly due to influencing glucose homeostasis through direct SGLT interaction, indirect metabolic pathways, or a combination of both. This review examines their molecular mechanisms, absorption profiles, and inhibitory activity on sodium–glucose cotransporters SGLT1 and SGLT2. While resveratrol demonstrates high cellular uptake and metabolic conversion, ε-viniferin exhibits poor intestinal permeability, suggesting limited systemic bioavailability but potential local activity at the intestinal epithelium. Gliflozins are clinically validated dual SGLT1/SGLT2 inhibitors that offer superior glucose-lowering efficacy and organ protection. In regard to stilbenoids, they offer promising in vitro results, though in vivo studies and clinical trials are scarce. Understanding resveratrol and viniferin pharmacokinetics, target interactions, and limitations is vital for their development as potential complementary or even alternative antidiabetic therapies.

## 1. Introduction

The global rise in diabetes mellitus presents a major public health challenge in humans but also in animals. It is estimated that, in Europe, 1.2% of dogs and 0.4% of cats will develop diabetes during their lifetime [1]. According to the International Diabetes Federation, more than 9% of people are currently diagnosed with diabetes in Europe [2]. Type 2 diabetes is considered to be the most prevalent form [3]. Although insulin secretion in affected individuals may range from deficient to excessive, the hormone is rendered ineffective by impaired tissue responsiveness [4]. At the same time, diabetes is one of the leading causes for cardiovascular and renal diseases—cardiovascular complications being the primary cause of mortality in the population suffering from this metabolic disease [3].

Modulating peripheral glucose metabolism by inhibition of intestinal absorption or by enhancement of renal glucose excretion, while employing counterregulatory mechanisms to restore metabolic balance, may present a promising approach for glycemic control [5]. SGLT1/2 inhibitor drugs like gliflozins (canagliflozin, sotagliflozin, dapagliflozin, empagliflozin, ertugliflozin) have recently been approved as a novel class of glycemic control agents [5]. These synthetic compounds are currently used for the treatment of diabetes mellitus, with proven effectiveness in managing this metabolic disease, while also offering additional benefits. Side effects are a common feature of synthetic drugs, in line with other medicinal agents; however, in case of gliflozins, their clinical benefits often outweigh these limitations [6,7,8].

Alongside synthetic drugs, plant-derived bioactive compounds like quercetin, kaempferol, epigallocatechin gallate, resveratrol and viniferin are gaining recognition in biomedical research. Amongst those, certain compounds are well studied, whereas others are comparatively under-investigated. Included in the latter category are resveratrol and viniferin, which offer a promising reservoir of compounds capable of selectively modulating intestinal and renal glucose transporters. Recent studies show that these molecules and their isoforms inhibit SGLT1 and/or SGLT2 glucose cotransporters [9,10,11,12,13]. In the literature, besides antidiabetic properties, numerous other beneficial effects of these compounds are mentioned [13,14].

This review aims to summarize and report the currently available findings on the inhibitory effects of resveratrol and viniferin with interest towards their therapeutic roles. While gliflozins are synthetic SGLT inhibitors already established as effective treatments for diabetes mellitus, we aim to underscore the possibility that these plant-derived compounds could, with further translational research, evolve into effective therapeutic options for diabetes management.

## 2. Materials and Methods

### 2.1. Literature Search Strategy

A comprehensive literature search was conducted across major scientific databases, including PubMed, Scopus, and Web of Science, using keywords such as “resveratrol”, “viniferin”, “SGLT1”, “SGLT2”, “gliflozin” and “glucose transport” in combination with “mechanism”, “structure” and “diabetes therapy”. Peer-reviewed articles published between 2000 and 2024 were screened for relevance based on predefined inclusion criteria focusing on molecular interactions, transporter biology, and pharmacological applications.

### 2.2. AI-Enhanced Analysis

Throughout the development of this review article, Windows AI assistant Copilot contributed to the synthesis of complex scientific literature by providing tailored summaries of research findings and conceptual overviews. Through the AI-assisted tool, the experimental designs and key insights were extracted. These contributions were guided and verified by the authors to ensure scientific accuracy and relevance, making the AI-assisted process both efficient and rigorously human-curated.

### 2.3. Operational Flow

Zotero was employed as a dedicated reference management tool to organize, annotate, and streamline the citation process. By facilitating the efficient collection of peer-reviewed articles and research data from multiple databases, Zotero enabled consistent documentation and cross-referencing of sources throughout the manuscript. Its integration with word processing software ensured precise in-text citations and a dynamically generated bibliography aligned with journal formatting guidelines.

This tool played a central role in maintaining scholarly rigor and traceability of the literature discussed.

### 2.4. Data Extraction and Validation

Data extraction involved cataloging findings related to resveratrol and viniferin structures and mechanisms of action, SGLT transporter function, and therapeutic outcomes in clinical and preclinical models.

To streamline analysis and improve consistency, AI-assisted tools were employed for text summarization, semantic mapping, and synthesis of key concepts across sources. These enhancements ensured rapid identification of thematic trends and cross-study comparisons. All AI-generated content was rigorously reviewed and validated by human oversight.

Through the fusion of AI-enhanced screening tools and expert assessment, this review rigorously explored the molecular frameworks of SGLT1/2, the pathways by which resveratrol and viniferin regulate glucose homeostasis, as well as antidiabetic therapeutic insights.

## 3. Results and Discussions

### 3.1. Sodium–Glucose Cotransporters in the Intestine and Kidney

#### 3.1.1. SGLT1

SGLT1 is a 664 amino acid membrane protein with 14 transmembrane α helices and both termini located extracellularly. Its C-terminal domain (TM10–TM14) contains two sugar-binding sites. The external binding site likely represents the high-affinity site, while the internal binding site might be the low-affinity site [15].

This protein is highly expressed on the brush border membrane of enterocytes in the small intestine, where it mediates glucose absorption. It is also present in the kidney (S2 [16] and S3 [17] segments), and has been detected in salivary glands and the heart [17].

Research shows that expression of this protein in the intestine increases with high glucose diets [18,19,20] and after luminal glucose infusion [18,19]. It has been reported that in mice, 24–48 h fasting enhances SGLT1 activity in the intestine [21]. Segment-specific studies done by Klinger and Breves (2018) show higher electrogenic glucose transport in the ileum compared to the jejunum, despite similar or lower SGLT1 protein levels [22].

The SGLT1 transport mechanism is described as a secondary active transport that uses the sodium gradient—maintained by the Na^+^/K^+^-ATPase pump— to facilitate glucose uptake. SGLT1 protein cotransports one D-glucose molecule together with two sodium ions into the cell [23,24]. The transport process involves firstly the binding of the two sodium ions to the protein, which increases the transporters’ affinity for glucose [15,23]. As a repercussion, one glucose molecule binds to the transporter. This event triggers conformational changes that will result in translocation of the glucose molecule, followed by that of the two sodium ions. SGLT1 then suffers another conformational change which allows the protein to expose the sodium- and glucose-binding sites towards the luminal side. Wright et al. (2004) mention that SGLT1 can recycle approximately 1000 times per second at 37 °C [15]. The sodium-dependent glucose transporter SGLT1 in the small intestine is characterized by low capacity and high affinity [15], as the approximate value of the substrate concentration at which the reaction rate reaches half of its maximum velocity is *Km* = 0.5 mM for D-glucose [23]. One study reports that, in humans, glucose *Km* is 2 mM/L for SGLT1 [15].

- In the literature, it is mentioned that the regulation of SGLT1 involves sweet taste receptors T1R2/T1R3 found on enteroendocrine cells, which activate a signaling cascade that leads to: increasing of enterocyte cAMP levels, stabilization of SLC5A1 mRNA, and upregulated insertion of SGLT1 into the apical membrane [19,25].
- Regulation of the SGLT1 transporter, as Sabino-Silva et al. (2010) describe, occurs through protein kinases through either: the direct pathway which implies the β-adrenoreceptors; the indirect regulation described as the modifications tied to the insertion and retrieval of the SGLT1 protein into the plasma membrane [17].

Other molecules transported by SGLT1, besides glucose, are D-galactose [23] and water [15,17,26]. Two distinct mechanisms have been reported regarding water absorption through SGLT1:

Cotransport of 200–260 water molecules per cycle with Na^+^ and glucose [17,26];

Passive water/urea flow through channels formed by the C-terminal C5 domain [27].

#### 3.1.2. SGLT2

The gene that is responsible for encoding the sodium-dependent glucose transporter SGLT2 is *SLC5A2* [17,28]. This protein is found mainly in the kidneys, namely in the initial segments of the proximal renal tubule [17]. In mouse kidneys, Park et al. (2018), cited by Ghezzi et al. (2018), report that in single-cell RNA sequencing experiments, SGLT2 is specifically and uniquely active in cells of the S1 tubular segment [28]. In man and mice, HNF-1α seems to directly control the renal expression of SGLT2 [29]. In diabetic rats, it is reported that both the level of SGLT2 and HNF-1α mRNA expression is 50% higher [30]. SGLT2 is also expressed in other tissues like intestine, liver, mammary glands, lungs, skeletal muscle, and spleen [17].

SGLT2 protein is made up of 672 amino acids. Structurally, this protein exhibits two termini, NH2 and COOH, which are positioned extracellularly [17]. The stoichiometry of SGLT2 is 1:1, namely one sodium ion is transported along one glucose molecule. Even though in this transporters’ structure two sodium-binding sites are present, the region where the second sodium ion would attach (Na3) is occluded [31].

Hiraizumi et al. (2024), using cryo-electron microscopy, revealed the outward-facing and inward-open structures of the human SGLT2 transporter during glucose transport [31]. The authors report that SGLT2 movement follows the alternating-access mechanism, while also mentioning its two components:

- the immobile fraction provides a fixed frame, made up of transmembrane helices: TM1, TM2, TM6, TM7, TM11, TM12 and TM13; this structure includes the bundle domain, which offers an anchor point for conformational changes during glucose transport, and, as the same researchers state, it is made up of transmembrane helices: TM1, TM2, TM6, TM7. Mentioned is also the protein MAP17, which aligns closely to TM13 stabilizing the bundle domain and the hash domain that rotates around the sodium-binding site, regulating the inward- and outward-facing conformations of SGLT2 [31].
- the mobile component is made up of transmembrane helices: TM0, TM3, TM4, TM5, TM8, TM9 and TM10; inward-open and outward-facing conformations are determined by movement of the gate helices, namely: TM5, TM10 [31].

SGLT2 is known to mediate the reabsorption of more than 90% of glucose from the kidney filtrate; however, its detailed functional characterization remains relatively limited [15,32]. In the last decade, studies regarding this domain have emerged, as whole-cell patch-clamp electrophysiology experiments on embryonic kidney 293T cells show a similar affinity for D-glucose of both SGLT1 and SGLT2 under physiological conditions in the straight proximal tubule [32], despite the classical view of SGLT1 as a high-affinity, low-capacity transporter and SGLT2 as a low-affinity, high-capacity one [15].

Hummel et al. (2011) indicate that when glucose transport is saturated, the maximum current can be recorded (*Imax*), while charge movements before transport starts are attributed to the pre-steady-state currents (*Qmax*) [32]. The researchers underlined that SGLT1 seems to have a 10 times greater *Imax* value than that of SGLT2, and that no *Qmax* was detected in SGLT2. The findings suggest that the two transporters differ fundamentally in their kinetic behavior, turnover, or membrane abundance, though it is unclear if the results obtained are due to greater copy numbers of the transporter, to a higher turnover rate, or both [32].

### 3.2. Inhibitors of Sodium–Glucose Cotransporters SGLT1 and SGLT2

In 1999 phlorizin, a natural compound extracted from apple bark was used in streptozotocin-induced diabetic rats. As a result, blood glucose levels decreased rapidly, with glucose being excreted through the urine. This natural compound was the first agent discovered to competitively inhibit SGLT1 and SGLT2 [5,33]. Glucoside is described as a specific, high-affinity, competitive inhibitor of sodium–glucose cotransporters 1 and 2. Phlorizin is made up of a glucose-like structure which binds to the SGLT C-terminal domain and a phloretin tail, which binds to an ectodomain, stabilizing the transporter in a non-functional state, therefore preventing structural changes that could lead to glucose transport [34]. Phlorizin is not transported across the membrane of the cells, it remains bound to the SGLT domain. Being a competitive inhibitor, at a certain luminal glucose concentration it is possible for this compound to be dislodged from the binding site [15,35]. Phlorizin is a potent inhibitor of SGLT1 and SGLT2, but certain adverse effects were also noted, as oral administration is associated with diarrhea and gastrointestinal disturbances [36], therefore limiting its clinical use.

In recent years, pharmacological inhibition of SGLT transporters has emerged as a therapeutic strategy for patients with Type 1 and 2 diabetes mellitus. SGLT1/2 synthetic inhibitors, including canagliflozin, dapagliflozin, empagliflozin and sotagliflozin, have shown great results in lowering blood glucose levels and promoting glucose excretion [37,38,39,40,41,42]. Their efficacy and safety are supported by extensive human trials [5,6,43,44]. Some studies report adverse effects during in vivo treatment with gliflozins [36,45]. However, Mulani et al. (2025) mention that even through there are certain side effects that might occur when gliflozins are administered, they are generally well-tolerated [8], and even provide additional benefits [6,7,8,46].

Gliflozins are currently used as a first-line treatment for diabetes, yet researchers continue to search for new therapeutic compounds. Increasing attention has turned toward plant-derived molecules, particularly those capable of modulating glucose homeostasis. These natural agents may directly inhibit SGLT transporters, indirectly influence them through metabolic signaling pathways, or in some cases combine both mechanisms [14,47,48,49,50]. Some of those plant-based substances are flavanones like (-)-kurarinone or sophoraflavanone G extracted from *Sophora flavescens*, indole alkaloids like alstiphyllanine D or 10-methoxy-N(1)-methylburnamine-17-O-veratrate extracted from *Alstonia macrophylla* and stilbenoids like viniferin and resveratrol extracted from *Vitis vinifera* [51]. Within this diverse group, resveratrol and its oligomeric derivative viniferin remain comparatively less studied, although several reports have examined their potential antidiabetic effects, while most of the available evidence is largely based on in vitro experiments. Their mechanism of action differs from that of gliflozins, highlighting the need to further investigate how these polyphenols influence glucose homeostasis. According to current research, these plant-derived polyphenols influence glucose homeostasis through various pathways: modulation of intracellular signaling, membrane interactions, and localized intestinal effects [10,11,47,52,53].

Assessment of the inhibitory effect of certain compounds towards SGLT1 and SGLT2 activity can be done through a number of techniques. Most research assessments are done via in vitro methods, while direct in vivo inhibition of SGLT1/SGLT2 by viniferin or resveratrol is not yet conclusively demonstrated [11,54,55]. However, the anti-hyperglycemic and anti-hyperlipidemic potential of these compounds is evaluated through blood glucose assessments, insulin levels, glucose transporters expression, total cholesterol, triglyceride value, low-density lipoprotein-cholesterol value and glucose tolerance tests [47]. In Table 1, methods used to assess the inhibitory activity of gliflozins, resveratrol, viniferin (or their isomers) on SGLT1 and/or SGLT2 transporters can be seen.

Resveratrol, also known as 3,5,4′-trihydroxy-trans-stilbene, is a phenolic compound that is produced by plants as a defense mechanism in response to certain stressors: microbial attack, toxins, infections or UV radiation [11]. First isolated in 1940, it is known to be abundant in grape skins, where the trans-isomer predominates and shows the highest stability and biological activity [59]. Its bioavailability is generally low, although encapsulation strategies (e.g., yeast, liposomes) can improve stability and absorption [55].

Absorption of resveratrol in the intestine occurs through diffusion or by forming complexes with certain membrane transporters [48]. Another possible pathway is transmembrane transport of resveratrol via raft-dependent endocytosis [22]. Biophysical studies show that resveratrol localizes near the interface of the hydrophilic headgroups and hydrophobic core of the lipid bilayer, which may contribute to its membrane-associated regulatory effects [60,61]. However, unlike gliflozins, its interaction with glucose transport seems to be indirect. In Ussing chamber experiments, resveratrol reduced glucose-induced short-circuit currents, suggesting decreased SGLT1-mediated glucose absorption [22], yet the evidence points towards regulatory effects, not direct transporter inhibition [10,22]. Studies attribute these changes to intracellular signaling pathways—such as AMPK activation—or to adaptive SGLT1 phosphorylation, rather than a direct binding interaction with the transporter [10]. To date, we could not find any molecular data that demonstrate direct competitive inhibition of SGLT1 by resveratrol. Most inhibitory effects of resveratrol reported in the literature arise from systemic actions [48,62,63].

After absorption, resveratrol binds to albumins and lipoproteins, which act as carriers and facilitate tissue uptake [62]. This compound has been detected in vascular tissues for up to 24 h after oral administration, and one study reports interactions with endothelial SGLT1, though the functional significance remains unclear [63]. Beyond glucose transport modulation, there are other benefits associated with resveratrol administration [64].

When resveratrol molecules undergo coupling through phenoxyl radical intermediates—a process first proposed by Langcake and Pryce (1977)—oligomeric forms of the compound are generated [11]. The bond can form at different positions on the aromatic rings, resulting in region-isomers. The molecules that are formed are structurally similar but spatially distinct dimers. One of those molecules is viniferin. Amongst the forms of viniferin mentioned are α-viniferin, β-viniferin, ε-viniferin, δ-viniferin, γ-viniferin, vitisin A and vitisin B [11]. Structurally, viniferin is rich in carbon and hydrogen, making it poorly water-soluble and highly sensitive to UV light. More than 90% of trans-ε-viniferin converts to its cis-form within 30 min of UVA exposure, after which it degrades into less active phenanthrenes. This degradation process happens in other stilbenes, such as resveratrol [65].

In vitro studies show that ε-viniferin is minimally absorbed across the intestinal epithelium. Ex vivo experiments using porcine jejunum and ileum demonstrate that ε-viniferin reduces glucose-induced short-circuit currents, indicating inhibition of SGLT1-mediated electrogenic glucose transport [52]. However, direct binding to SGLT1 has not been conclusively demonstrated. Additional evidence from murine-everted gut sacs shows that 0.1 mM ε-viniferin reduces α-methyl-glucopyranoside uptake by 50%, and data suggest a non-reversible inhibitory effect [54]. One proposed mechanism is that ε-viniferin interacts with membrane phospholipids, rather than binding directly to the transporter [34,54]. However, molecular docking studies suggest that ε-viniferin potentially blocks glucose access to SGLT2 [66].

In vivo studies indicate broader metabolic benefits associated with viniferin administration. Mechanistically, ε-viniferin interacts with AMP-activated protein kinase (AMPK), binding residues in both α- and β-subunits, suggesting a role in AMPK activation and improved metabolic regulation. The lack of side effects observed during the mentioned study could suggest a favorable tolerability of this compound at doses up to 60 mg/kg [47]. Studies report additional antilipidemic effects [14,47].

Table 2 shows a summary of synthetic gliflozins and natural compounds resveratrol and viniferin specification.

## 4. Conclusions

SGLT1 is expressed mainly in the small intestine, while SGLT2 is mostly expressed in the renal tubule. There are several factors that influence the level of their expression.

The sodium-dependent glucose-active transport mediated by SGLT1 and SGLT2 involves a multitude of cellular mechanisms. A better understanding of those proteins’ functional role might offer certain clinical insights regarding their modulation.

Phlorizin, while the first natural inhibitor of SGLT1/2 discovered, has limited clinical applicability.

Gliflozins represent a major advancement in antidiabetic therapy, offering selective SGLT1/2 inhibition with proven efficacy in glycemic control and cardiovascular protection.

Viniferin and resveratrol offer natural, multi-targeted support in diabetes and other metabolic states related to diabetes. The mechanisms involved in glucose regulation mediated by stilbenoids are complex. SGLT’s direct inhibitory mechanism is yet to be subjected to further studies. While resveratrol and viniferin inhibition of glucose uptake is supported by functional data, molecular studies that explicitly characterize the direct inhibitory interaction between those polyphenols and SGLT are necessary.

Comparative analysis between synthetic gliflozins and the natural compounds viniferin and resveratrol reveals certain advantages and limitations regarding their use as antidiabetic agents. Synthetic gliflozins show great clinical efficacy and additional beneficial effects, while also being associated with certain health risks. The plant-derived compounds seem well-tolerated but limitations due to poor bioavailability and rapid metabolism should be taken into consideration, as those pharmacokinetic barriers challenge systemic therapeutic effects. Moreover, the lack of clear in vivo evidence for direct SGLT1/2 inhibition, together with the absence of clinical studies, stand out as a major gap that requires subsequent research investigation.

Future studies should focus on clarifying the molecular interactions between natural stilbenoids and SGLT transporters in order to establish a better understanding of their mechanistic contributions to glucose transport modulation potential.

## Figures and Tables

**Table 1:** Methods used for assessment of gliflozins’, resveratrol’s or viniferin’s inhibitory effect on SGLT1/2.

Method Type	Description	Agent Tested	Dose	SGLT1 IC_50_	SGLT2 IC_50_	Results	Source
In vitro	Uptake assay of AMG ^2^ on JTREx-293-SGLT1 cells	canagliflozin	20 μM extracellular application	1550 nM	6.8 nM	almost complete SGLT1 and SGLT2 inhibition	[56]
	Uptake assay of AMG ^2^ on JTREX-CHO-SGLT2 cells		20 μM–200 nM intracellular application			no significant inhibition showed	
In vitro	[^14^C]-AMG ^2^ uptake assays on stable cell lines over-expressing human SGLT-1, 2	empagliflozin	0.01 nM–100 µM	8300 nM ^3^	3.1 nM ^3^	inhibition increases with increasing glucose concentrations for hSGLT1, only partial inhibition at 100 µM empagliflozin	[57]
						potent and highly selective inhibitor of hSGLT2	
In vitro	Use of Chinese hamster ovary K1 wild-type cells expressing human SGLT1/SGLT2 to measure uptake of fluorescent glucose analog 1-NBD-glucose	(+)-ε-viniferin	-	58 μmol/L ^1^	110 μmol/L ^1^	44% inhibition of SGLT1, little inhibition of SGLT2	[58]
		(-)-ε-viniferin	-	-	-	no inhibition of SGLT1, 35% inhibition of SGLT2	
In vitro/Ex vivo	Measurement of electrogenic glucose transport across porcine intestinal epithelium (jejunum and ileum) in the Ussing chamber	ε-viniferin	0.3 mM	-	-	50% decrease in glucose-induced short-circuit current (SGLT1)	[52]
		trans-resveratrol	0.3 mM	-	-	50% decrease in glucose-induced short-circuit current (SGLT1)	
In vitro	Measurement of sodium-dependent glucose uptake at the cellular membrane level using brush-border membrane vesicles	ε-viniferin	0.3 mM	-	-	almost complete inhibition of sodium-dependent D-glucose uptake (SGLT1)	
In vitro	Measurement of [^14^C] α-MDG uptake into everted gut rings of isolated intestinal segments from C57BL/6N mice	trans-resveratrol	0.3 mM	-	-	40–50% inhibition of sodium-dependent D-glucose uptake (SGLT1)	[54]

^1^ expressed as mean values; ^2^ α-Methyl-D-glucopyranoside; ^3^ human SGLT1/2.

**Table 2:** Summary of synthetic gliflozins and natural compounds resveratrol and viniferin specifications.

Type of Compound	Synthetic				Natural	
Specifications	Canagliflozin	Dapagliflozin	Empagliflozin	Sotagliflozin	Resveratrol	Viniferin
**Type of inhibition and target transporter**	direct potent SGLT2 inhibitor; weak SGLT1 inhibition [43]	direct potent and selective hSGLT2 ^3^ inhibitor [37,38]	direct potent SGLT2 inhibitor [37]	direct dual inhibitor: SGLT1 and SGLT2 [8,37]	indirect inhibitor of SGLT1 [10,48,62,67]	binds to membrane phospholipids and disrupts the conformational flexibility of SGLT1 [34]; might act as a direct, non-reversible inhibitor of SGLT1 [54]; direct inhibition of SGLT2—binds to the Q457 residue of transporter [66]
**IC_50_**	SGLT2 IC_50_ = 4.2 nM; SGLT1 IC_50_ = 660 nM [43]	hSGLT2 ^3^ EC_50_ = 1.1 nM ^4^ [37]	SGLT2 IC_50_ = 3.1 nM [37]	SGLT1 IC_50_ = 36 nM; SGLT2 IC_50_ = 1.8 nM [37]	hSGLT1 ^3^ IC_50_ = 510 ± 90 μM [54]	hSGLT1 ^3^ IC_50_ = 8.0 ± 0.48 μM; SGLT1 IC_50_ = 34.8 ± 9.7 μM [54]
**Clinical Status**	approved for T2DM ^2^ [36,46]	approved for T2DM ^2^ [8]	approved for T2DM ^1^ [45]	approved for treatment of T1DM ^1^ & T2DM ^2^, heart failure, chronic kidney disease [8]	scarce clinical data/no assessment for clinical use	scarce clinical data/no assessment for clinical use
**Benefits**	glycemic control; possible neuroprotective [46]	glycemic control; cardiovascular and renal benefits [8]	glycemic control; cardiovascular benefits [8]; long-term treatment may enhance metabolic syndrome outcomes in diabetic rats [39]	improved glycemic control in T2DM ^2^ patients [6]; risk of diabetic ketoacidosis due to fasting-like state induced by dual SGLT inhibition [6]; reduced postprandial blood glucose peak [6,7]; improved cardiovascular function [7]	improved glycemic control [48]; protection against lipid hydrolysis [60,61]; enhances intestinal barrier integrity disrupted by hyperglycemia [64]	reduced body weight gain, decreased adipose tissue mass and lowered hepatic triglyceride levels along decreased leptin and insulin plasma concentrations [45]; reduced lipid accumulation in embryo fibroblast cells [45]; suppresses inflammatory markers [45]; reduce adipogenesis [45]; decreased serum levels of triglycerides, cholesterol and low-density lipoprotein cholesterol in diabetic rats [47]; improved glycemic control, improved blood glucose tolerance test results, inhibits pancreatic α amylase, potentially reducing postprandial hyperglycemia [53]
**Adverse Effects**	might increase occurrences of osmotic diuresis and volume depletion [36]; might increase risk of diabetic ketoacidosis [45]	might increase risk of diabetic ketoacidosis [45]	might increase risk of bladder cancer [45]	might increase risk of diarrhea, genital fungal infections, urinary tract infections [8]; might increase risk of hypoglycemia if not associated with insulin therapy [6,42]	scarce clinical data/not reported	scarce clinical data/not reported
**Pharmacological Features**	high bioavailability [37]	high bioavailability [38]	moderate to low bioavailability in rats; high bioavailability in dogs [39]; broad selectivity (SGLT4/5/6); long binding half-life [57]	high bioavailability [8]	rapid metabolism [48], low bioavailability [68]	might be deposited in white adipose tissue after administration [40]; low bioavailability [40,65];
**Distinctive attributes**	dual activity: antidiabetic and potential Alzheimer’s benefits [46]	multi-target agent: beneficial effects in regard to glycemic control, cardiovascular and renal function [8]	dual activity: antidiabetic and cardiac protection [57]	dual beneficial activity: improved glycemia control, improved cardiovascular function [6,7]; safe clinical profile if patient is monitored and practices good personal hygiene [8];	multi-target agent: antioxidant, relatively well-tolerated during the in vivo study [14]; intestinal barrier restorative effects [64];	multi-target agent: antidiabetic [11]; anti-inflammatory [41]; relatively well-tolerated during in vivo studies [14,47]; antilipidemic [41,47]

^1^ Type 1 diabetes mellitus; ^2^ Type 2 diabetes mellitus; ^3^ human SGLT; ^4^ concentration at which the drug reaches 50% of its maximal biological effect.

## Data Availability

No new data were created or analyzed in this study.

## Abbreviations

The following abbreviations are used in this manuscript:

SGLT1	sodium dependent glucose active transporter 1
SGLT2	sodium dependent glucose active transporter 2
Imax	maximum current
Qmax	pre-steady-state currents
Km	glucose concentration at which the transport velocity is one-half its maximal value
IC_50_	half maximal inhibitory concentration
EC_50_	concentration at which the drug reaches 50% of its maximal biological effect
AMP	adenosine monophosphate
AMPK	adenosine monophosphate activated protein kinase
Ki	inhibition constant
